# Contact Patterns Drive Age-Structured Transmission Dynamics and Seasonality of Scarlet Fever

**DOI:** 10.3390/pathogens15030296

**Published:** 2026-03-09

**Authors:** Jing He, Jijun Zhao

**Affiliations:** Institute of Complexity Science, College of Automation, Qingdao University, Qingdao 266071, China; hejing@qdu.edu.cn

**Keywords:** *Streptococcus pyogenes*, scarlet fever, age-structured SIR model, contact patterns, time-varying reproduction number

## Abstract

Background: Scarlet fever has seen a sharp increase in its reported incidence in China since 2011, and this study focuses on Shanghai as a representative setting to systematically investigate its transmission dynamics by analyzing age structure. It further identifies high-risk age groups and provides a theoretical foundation for prevention and non-pharmaceutical intervention strategies. Methods: We developed an SIR model that incorporates age structure and seasonality of transmission rate. In parameter estimation, the methodology of the partially observed Markov process framework is employed to derive results based on monthly data. The time-varying reproduction number $R_{0}\left(t\right)$ is derived monthly from the next-generation matrix. Age-specific forces of infection are estimated to identify high-risk groups and quantify how school-term-driven contact patterns modulate transmissibility. Results: The force of infection peaked in children aged 7–9 years, whereas the force of infection was highest among adults aged 35–39 years. The seasonal amplitude for transmission among school-aged groups was 39% (95% CI: 37–41%). The estimated $R_{0}\left(t\right)$ varied seasonally between 3.02 and 8.83. Conclusions: The transmission rate in Shanghai shows strong age heterogeneity and school-driven seasonality. Children aged 7–9 years are the highest-risk group, and interventions should target them during periods of high $R_{0}\left(t\right)$.

## 1. Introduction

Scarlet fever is an acute respiratory infectious disease [1] caused by group *A Streptococcus* [2], mainly transmitted through respiratory droplets [3]. Infection may lead to a series of severe complications, such as rheumatic fever and nephritis, posing long-term challenges to the public health system [4]. In the past centuries, it was one of the most common childhood infections worldwide. With the widespread use of antibiotics and improvements in sanitation and nutrition in the 20th century, its incidence declined markedly before stabilizing [3]. Since 2011, several countries in Asia and Europe have reported a resurgence of scarlet fever [5,6,7], and China has also observed a notable increase during the same period [8]. Given Shanghai’s high population density, elevated reported incidence of scarlet fever, and the availability of a published social contact survey that provides an essential contact-rate matrix [9], this study selected Shanghai as the analytical setting. From 2011 to 2024, Shanghai reported 25,539 scarlet fever cases, with an annual incidence of 12.7 per 100,000 people, representing a persistent public health threat [10]. Currently, no effective vaccine is available for its prevention [11]. Scarlet fever incidence exhibits marked age-dependent variation, with the highest burden consistently observed in preschool and school-aged children [4]. This pattern is primarily attributed to immunological naivety in young populations, frequent close contact in school and kindergarten settings, and consistent epidemiological evidence from multi-country data showing peak incidence in children aged 5–12 years [5,7]. Understanding these mechanisms is essential for developing age-structured transmission models and informing targeted intervention strategies.

Current research on scarlet fever focuses mainly on its epidemiological and spatiotemporal characteristics in China [1,3,12], which are usually based on a descriptive analysis of the age and sex distribution of cases, together with the observed periodicity and spatial clustering. To better understand disease risk and design effective control strategies, a deeper analysis of its transmission dynamics is required. This requires estimating three key parameters: the force of infection (λ), reflecting the per capita risk of infection [13]; the transmission rate (β), defined as the transmission probability per unit time, whose periodic variation drives seasonality [14]; and the basic reproduction number ($R_{0}$), representing the average secondary cases caused by an infected individual in a fully susceptible population [15]. Accurate estimation of these parameters is crucial for understanding outbreak scale, periodicity and informing control interventions. Comparative studies have shown that the peak force of infection occurs at different ages for different diseases—around age 6 for pertussis and below age 1 for measles [13,16]—but comparable analyses are lacking for scarlet fever. Domestic studies typically report values between 1.02 and 1.8, while a nationwide analysis estimated $R_{0}$ at approximately 3.56 [17]. The latter estimate is closer to values reported for similarly transmitted infections such as mumps and aligns better in magnitude with pre-vaccination era estimates for measles and pertussis (10–18) in Western countries [14,15].

**Figure 1:**
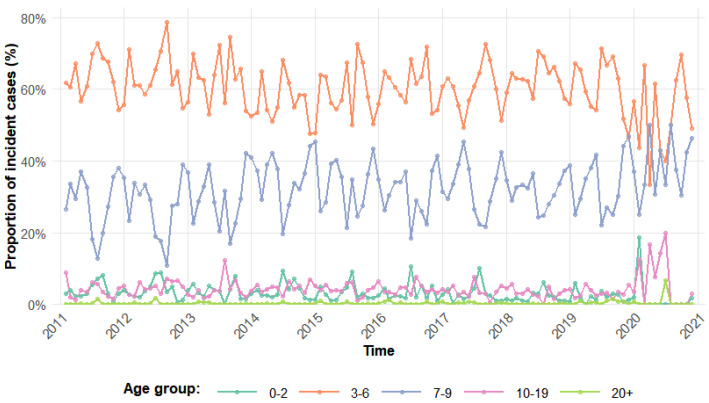
Proportion of scarlet fever cases by age group in Shanghai, 2010–2020, aggregated to the five age groups used in the transmission model.The bar chart displays the age distribution of 25,539 notified scarlet fever cases over the study period. Cases were grouped into five epidemiologically relevant categories: 0–2, 3–6, 7–9, 10–19, and ≥20 years, to align with the transmission model structure. The 3–6 and 7–9 year groups together account for over 70% of all cases, with the highest proportion in the 7–9 year group (∼40%). Adolescents (10–19 years) contribute ∼15%, while adults (≥20 years) represent <5% of cases, with cases in those aged ≥60 years being negligible (<0.1%).

Understanding infectious disease transmission requires dynamical models. For scarlet fever, studies have used SIR, SEIQR, and TSIR models to estimate transmission rate and $R_{0}$ [11,17,18]. However, most studies have not adequately considered the heterogeneity of both population age structure and contact patterns. As a childhood disease, its incidence varies strongly by age (Figure 1), suggesting that the force of infection and transmission rate likely differ across age groups. Therefore, age structure should be included in the model [14], although no such model has been developed for scarlet fever, lacking a comprehensive framework that can link observed risk patterns with contact patterns. Many childhood infectious diseases exhibit seasonal variation in transmission rates [11]. For instance, studies on varicella, pertussis, and measles have shown that periodic changes in contact rates among school-aged populations directly drive the seasonality of transmission [13,16,19]. Shanghai, as a key epidemic area, provides a basis for quantifying local age-specific contact patterns and investigating their driving role through its published social contact survey data [9]. Therefore, a compartmental model that simultaneously integrates age-specific contact patterns and the seasonality driven by changes in such contact patterns is essential for understanding the transmission dynamics of scarlet fever. Given the short incubation period of scarlet fever (averaging 2–3 days [17]), this study adopts the SIR model as the core framework, aiming to construct a contact-pattern-driven age-structured model to elucidate the age heterogeneity in scarlet fever transmission and the contact-rate-driven seasonal mechanisms.

To characterize the dynamics of scarlet fever transmission and estimate its key parameters, an age-structured compartmental model with seasonal forcing is required. Using Shanghai as a case study, this work estimates the age-specific force of infection to identify high-risk groups, develops an age-structured seasonally forced transmission model to quantify how school-term-driven contact patterns modulate transmission rate seasonality, and applies the next-generation matrix method to estimate $R_{0}$.

## 2. Materials and Methods

### 2.1. Materials

Reported scarlet fever cases in Shanghai (2011–2020), stratified by age, were obtained from the Chinese Center for Disease Control and Prevention [20] (https://www.chinacdc.cn/). Age was grouped into 26 categories: single years for ages 0–9, 5–year intervals from age 10 onward, with the last group comprising individuals aged 85 years or older. Shanghai’s birth rate, mortality rate, and total population were obtained from the Shanghai Municipal Bureau of Statistics [21].

### 2.2. FOI Estimation Framework

Age-specific force of infection (FOI) can typically be estimated using either reported case counts or serological data. Due to the lack of detailed serological data for scarlet fever in China, this study employed reported case counts for estimation. The data used were derived from the notified scarlet fever cases in Shanghai from 2011 to 2020, as described in Section 2.1. The original age stratification included single-year age groups for ages 0–9 years, 5–year intervals for ages 10 years and above, with individuals aged 85 years and over forming the final group. Given that reported cases among individuals aged 60 years and above were extremely low (approaching zero), this population was aggregated into a single age group. Consequently, FOI was calculated only for the first 20 age groups (corresponding to ages 0–59 years, following the same grouping scheme as above), which for the *i*-th age group can be expressed as [13,16]:

(1) $$\lambda_{i}=-\frac{1}{\Delta a_{i}}Ln\frac{1-h_{i}}{1-h_{i-1}},$$

where $\Delta a_{i}$ is the width (age span) of the *i*-th age group, and $h_{i}$ represents the cumulative proportion of reported cases up to age group *i* relative to the total number of reported cases.

### 2.3. Age-Structured SIR Model for Scarlet Fever Incorporating Seasonal Variation

The choice of model structure is guided by data resolution and disease biology. While Susceptible–Exposed–Infectious–Recovered (SEIR) models are common, scarlet fever’s short incubation (2–3 days) is negligible relative to our monthly case data. An exposed compartment would not improve fit and could hinder parameter identifiability. Our focus is on quantifying age-structured, contact-driven seasonality, which the SIR framework captures efficiently. Taking into account the variation in FOI in Shanghai and the grouping structure of the contact matrix used [9], the population was regrouped into five categories: 0–2 years (toddlers with frequent household contact), 3–6 years (preschool children interacting mainly with family and kindergarten peers), 7–9 years (children exhibiting peak FOI), 10–19 years (adolescents with high peer-contact rates), and a single group of adults aged 20 years and above (combined because of the very low reported incidence in adults). Within each age group, individuals are classified into three compartments: susceptible (S), infected (I), and recovered (R). The model is described by the following equations:

(2) $$\left\{\begin{array}{c} \frac{dS_{i}}{dt}=\nu_{i}-\sum_{j}\beta_{ij}I_{j}S_{i}-\mu_{i}S_{i}+\alpha_{i-1}S_{i-1}-\alpha_{i}S_{i},\\ \frac{dI_{i}}{dt}=\sum_{j}\beta_{ij}I_{j}S_{i}-\gamma_{i}I_{i}-\mu_{i}I_{i}+\alpha_{i-1}I_{i-1}-\alpha_{i}I_{i},\\ \frac{dR_{i}}{dt}=\gamma_{i}I_{i}-\mu_{i}R_{i}+\alpha_{i-1}R_{i-1}-\alpha_{i}R_{i}, \end{array}\right.$$

where i=1,…,5. $\nu_{i}$ represents the birth rate. Newborns enter the 0–2 year age group, therefore, $\nu_{1}$ is set to the overall birth rate, while $\nu_{i}=0$ for i≠1. $\mu_{i}$ denotes the mortality rate of the *i*-th age group. It is assumed that only the fifth age group experiences nonzero mortality, equaling the actual death rate, while $\mu_{i}=0$ for all other age groups i≠5. $\alpha_{i}$ represents the rate of aging (age progression), describing the transfer of individuals from younger to older age groups. The first age group has no inflow from a younger group, and the fifth group has no outflow, $\alpha_{0}=0$ and $\alpha_{5}=0$. $\beta_{ij}$ is the transmission rate between susceptible individuals in age group *i* and infectious individuals in age group *j*, accounting for both within-group and between-group infections.

The average annual birth rate in Shanghai from 2010 to 2020 was 7.47‰ per year, and the average annual mortality rate was 8.54‰ per year. Assuming constant monthly birth and death rates, the corresponding parameters were set to ν=0.00062 per month and μ=0.00071 per month. Under the assumption of a uniformly distributed population, the aging rates between age groups were specified as follows: $\alpha_{1}=1/3,\;\alpha_{2}=1/4,\;\alpha_{3}=1/3,\;\alpha_{4}=1/10$. Aging is implemented as a discrete event at the beginning of each simulation year. Specifically, at the start of each year, individuals who have reached the lower age bound of the next age group are transferred in bulk according to the corresponding aging rate $\alpha_{i}$. Thus, $\alpha_{i}$ represents the annual proportion of the age group transferred and is set as the reciprocal of the residence time in that group (e.g., $\alpha_{1}$ = 1/3 per year for the 0–2 years group, which has a 3–year residence time). This formulation realistically captures the discrete nature of aging while avoiding additional assumptions about within-group age distributions. The parameter γ denotes the recovery rate. Given that the average recovery time for scarlet fever is 6.25 days [17], γ equals the reciprocal of the recovery period (Table 1).

**Figure 2:**
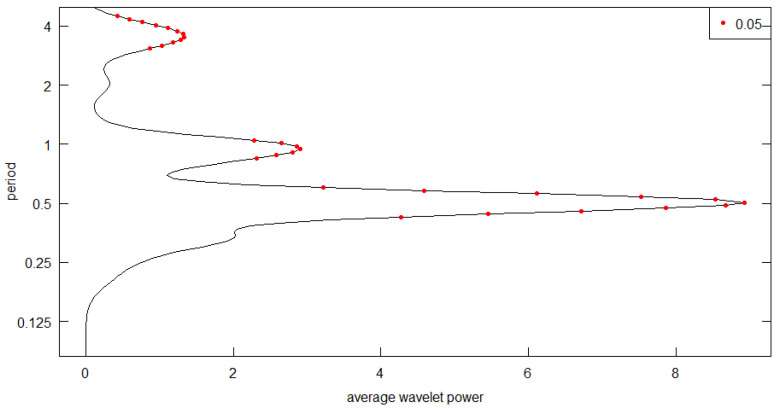
Average wavelet power spectrum of scarlet fever incidence from 2011 to 2020 (significance level α = 0.05). *X*-axis: average wavelet power; *Y*-axis: period (years). The black curve denotes the power distribution, and red dots represent significant power peaks at the α = 0.05 level. A significant periodicity with a dominant period of 0.5 years was observed.

In studies of seasonal transmission dynamics, two predominant forcing patterns are frequently employed: term-time forcing and sinusoidal forcing [14,15]. Surveillance data for scarlet fever exhibit a distinct semi-annual periodicity, with two incidence peaks occurring within each calendar year. Compared to term-time forcing, a cosine-based forcing formulation offers greater flexibility in capturing two closely coupled seasonal peaks over a single annual cycle (Figure 2).

Furthermore, existing evidence indicates that scarlet fever transmission rates peak in February and September each year [11]. This bimodal pattern necessitates the incorporation of a phase-shift parameter within the seasonal component. Relative to a standard sinusoidal forcing, the cosine forcing function facilitates more direct and precise alignment of the modeled peaks with these specific calendar months. Consequently, in the present study, we adopt a cosine seasonal forcing formulation. A seasonal component with a phase shift of 0.1667 is integrated into the transmission rate for the school-aged children and adolescent cohort. The phase shift δ = 0.1667 (corresponding to 2 months) aligns the peaks of the cosine forcing function with the estimated timing of peak transmission. Wavelet analysis (Figure 2) shows that scarlet fever incidence peaks in May–June and November–December; accounting for the delay between infection and case reporting (approximately 1 month), the transmission rate itself is expected to peak in March and September. Setting δ = 0.1667 shifts the cosine peaks from January and July to March and September, consistent with this epidemiological reasoning. For conciseness, define Y=k·cos (4π(t−0.1667)/12). For the school-aged groups *i* = 2, 3, 4, their within-group transmission rate is given by $\beta_{ii}^{*}\left(t\right)=\beta_{ii}\left(1+Y\right)$, where *k* scales the seasonal amplitude and is estimated later. We construct a transmission rate matrix that incorporates cosine-based seasonal forcing factors:

(3) $$\beta =\left[\begin{array}{ccccc} \beta_{11} & \beta_{12} & \beta_{13} & \beta_{14} & \beta_{15}\\ \beta_{21} & \beta_{22}\left(1+Y\right) & \beta_{23} & \beta_{24} & \beta_{25}\\ \beta_{31} & \beta_{32} & \beta_{33}\left(1+Y\right) & \beta_{34} & \beta_{35}\\ \beta_{41} & \beta_{42} & \beta_{43} & \beta_{44}\left(1+Y\right) & \beta_{45}\\ \beta_{51} & \beta_{52} & \beta_{53} & \beta_{54} & \beta_{55} \end{array}\right]$$

**Figure 3:**
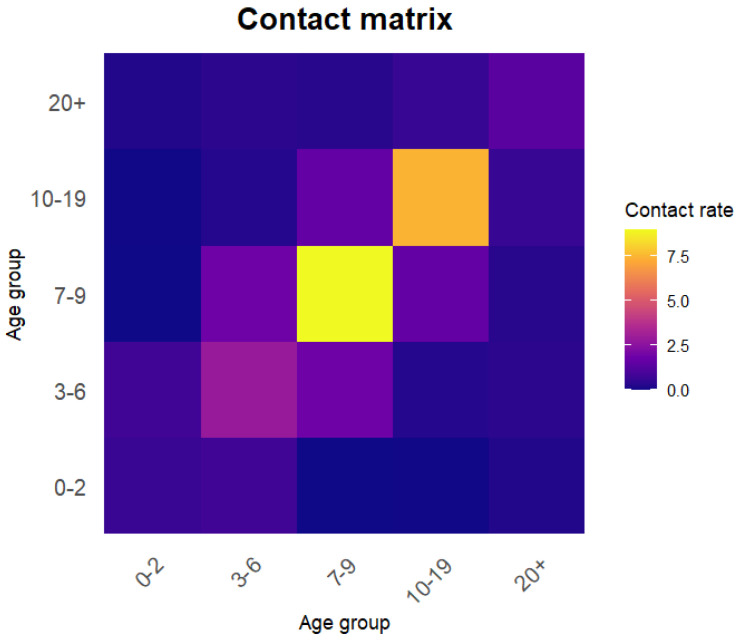
The contact-rate matrix partitioned according to the grouping in this study, reaggregated to the five age groups used in the transmission model (0–2, 3–6, 7–9, 10–19, and ≥20 years). Contact rates $c_{ij}$ represent the average number of contacts per day that an individual in age group *i* (rows) has with individuals in age group *j* (columns). The matrix is derived from a social contact survey conducted in Shanghai [9], using a uniform population distribution assumption within each original age stratum to compute weighted averages. Darker colors indicate higher contact rates.

The transmission-rate matrix is expressed as β = $q\cdot c_{ij}$, with *q* being the per-contact infection probability (to be estimated) and $c_{ij}$ the contact-rate matrix. The contact matrix $c_{ij}$ from Shanghai [9] was adopted for further analysis. In the cited literature, the contact matrix is originally stratified into 17 age groups: 0–2 years, 3–6 years, 7–9 years, 5–year intervals from 10 to 75 years, and a final group aged above 75 years. Assuming a uniformly distributed population, a weighted averaging method was applied to reaggregate the contact rates according to the age grouping used in this study, resulting in a 5 × 5 contact matrix (Figure 3).

### 2.4. Next-Generation Matrix Method

The next-generation matrix (NGM) method is a mathematical tool used to derive $R_{0}$, specially for age-structured compartmental models [14]. Linearizing the system at the disease-free equilibrium (DFE), the next-generation matrix is constructed, and $R_{0}$ is obtained as its spectral radius—that is, the largest eigenvalue of the matrix. The next-generation approach decomposes the infection process into two components: (1) the generation of new infections, represented by the matrix *F*, which describes the transition of susceptible individuals to the infected state; (2) the transfer of individuals, represented by the matrix *V*, which includes the recovery, death, and aging-driven movement of infected individuals between age groups.

At DFE, the system is linearized to derive the two matrices *F* and *V*, which correspond to the generation of new infections and the transition of individuals, respectively. An element $F_{ij}$ of matrix *F* represents the rate at which an infected individual in the age group generates new infections entering the age group within a fully susceptible population. The expression is: $F=\frac{\partial \left(\lambda_{i}S_{i}\right)}{\partial I_{j}}{|}_{DFE}$. The matrix can be written as follows:

(4) $$F=\left[\begin{array}{ccccc} S_{1}*\beta_{11} & S_{1}*\beta_{12} & S_{1}*\beta_{13} & S_{1}*\beta_{14} & S_{1}*\beta_{15}\\ S_{2}*\beta_{21} & S_{2}*\beta_{22} & S_{2}*\beta_{23} & S_{2}*\beta_{24} & S_{2}*\beta_{25}\\ S_{3}*\beta_{31} & S_{3}*\beta_{32} & S_{3}*\beta_{33} & S_{3}*\beta_{34} & S_{3}*\beta_{35}\\ S_{4}*\beta_{41} & S_{4}*\beta_{42} & S_{4}*\beta_{43} & S_{4}*\beta_{44} & S_{4}*\beta_{45}\\ S_{5}*\beta_{51} & S_{5}*\beta_{52} & S_{5}*\beta_{53} & S_{5}*\beta_{54} & S_{5}*\beta_{55} \end{array}\right]$$

where $\beta_{ij}$ is the transmission-rate matrix and $S_{i}$ is the proportion of susceptibles in age group *i*. Assuming a uniformly distributed population, these proportions are set as: $S_{1}=0.04,\;S_{2}=0.05,\;S_{3}=0.04,\;S_{4}=0.11,\;S_{5}=0.76$. The elements of the transition matrix describe the rates at which individuals leave the infected compartment (through recovery, death, or aging) and enter from other compartments. The diagonal entries represent the total outflow rate from each infected compartment, while the negative sub-diagonal entries represent inflow due to aging:

(5) $$V=\left[\begin{array}{ccccc} \alpha_{1}+\gamma_{1} & 0 & 0 & 0 & 0\\ -\alpha_{1} & \alpha_{2}+\gamma_{2} & 0 & 0 & 0\\ 0 & -\alpha_{2} & \alpha_{3}+\gamma_{3} & 0 & 0\\ 0 & 0 & -\alpha_{3} & \alpha_{4}+\gamma_{4} & 0\\ 0 & 0 & 0 & -\alpha_{4} & \mu_{5}+\gamma_{5} \end{array}\right]$$

Following the approach in [15], the basic reproduction number is calculated from the *F* and *V* matrices as:

(6) $$R_{0}=\rho \left(FV^{-1}\right),$$

where ρ(·) denotes the spectral radius, the largest eigenvalue of the matrix. It should be noted that the classical next-generation matrix method typically assumes the transmission system is in a steady state and uses a time-invariant constant transmission rate matrix β to compute $R_{0}$, resulting in a single numerical value [15]. However, in this study, the transmission rate β is explicitly modeled as a time-varying function driven by school terms. To quantify the dynamic changes in transmission potential over time, we introduce the concept of a time-varying basic reproduction number $R_{0}\left(t\right)$. Consequently, the $R_{0}$ values reported in this study essentially represent the monthly estimates of $R_{0}\left(t\right)$ during the study period from 2011 to 2020, whose seasonal fluctuations directly reflect the seasonal variation in β. By substituting the estimated parameters *q* and *k* into β, the basic reproduction number for scarlet fever in Shanghai is calculated.

### 2.5. Parameter Estimation and Model Fitting

This study employs the R (v4.5.1) package ‘pomp’ to construct a partially observed Markov process (POMP) framework [22], aiming to estimate two core parameters: the per-contact infection probability *q* and the amplitude of seasonal variation *k*.

First, the age-structured SIR dynamic model with five age groups (Equation (2)) is defined as the main process. The observation process links the model’s predictions to the monthly reported case data. Specifically, we assume that the reported new cases in age group *i* during month *t*, denoted $C_{ij}^{obs}$, follow a negative binomial distribution [23]:

(7) $$C_{i,j}^{\mathrm{obs}}∼\mathrm{NegBinom}\left(\mu =C_{i,j}^{\mathrm{sim}}\left(q,k\right),\;\psi \right),$$

where $C_{ij}^{obs}\left(q,k\right)$ is the corresponding monthly number of new infections simulated from the SIR model (Equation (2)) given parameters *q* and *k*, and ψ is an overdispersion parameter accounting for variability beyond the Poisson mean.

Parameter estimation was performed via maximum likelihood, combining particle filtering (sequential Monte Carlo, with 10,000 particles) with the iterative filtering (IF2) [24] algorithm. The particle filter approximates the conditional likelihood $L(q,k;C^{\mathrm{obs}})$ of the parameters given the observed data $C^{obs}$ by recursively updating the joint distribution of the latent states $X_{t}=\left(S_{t},I_{t},R_{t}\right)$:

(8) $$p\left(X_{t}|C_{1:t}^{\mathrm{obs}};q,k\right)\propto p\left(C_{t}^{\mathrm{obs}}|X_{t};q,k\right)\int p\left(X_{t}|X_{t-1};q,k\right)p\left(X_{t-1}|C_{1:t-1}^{\mathrm{obs}};q,k\right)dX_{t-1}.$$

The IF2 algorithm treats parameters as latent variables subject to Gaussian perturbations, iteratively refining them to maximize the log-likelihood. At each iteration *m*, parameters are perturbed as:

(9) $$\theta_{m}=\theta_{m-1}+\sigma_{m}\eta_{m},\;\eta_{m}∼N\left(0,I\right),$$

where θ=(q,k), $\sigma_{m}$ is a decreasing sequence of perturbation scales, and $\eta_{m}$ is a noise vector. The particle filter is used to evaluate the likelihood for each perturbed parameter set, guiding the search toward the optimum.

Uncertainty in the estimates was quantified using the likelihood profile method [25]. For each parameter, a profile log-likelihood $\ell_{p}\left(\theta_{j}\right)$ was computed by maximizing over the other parameter:

(10) $$\ell_{p}\left(\theta_{j}\right)=\max_{\theta_{\setminus j}}\;\log L\left(\theta_{j},\theta_{\setminus j};C^{\mathrm{obs}}\right),$$

from which the 95% confidence intervals for *q* and *k* were derived based on the chi-squared approximation with one degree of freedom ($\chi_{1,0.95}^{2}\approx 3.84$), i.e., $\left\{\theta_{j}:2\left[\ell_{\max}-\ell_{p}\left(\theta_{j}\right)\right]<\chi_{1,0.95}^{2}\right\}$. This follows from the asymptotic result that twice the log-likelihood difference between nested models differing by one parameter converges in distribution to a $\chi^{2}$ random variable under the null hypothesis. It should be emphasized that both parameters, *k* (seasonal amplitude) and *q* (probability of infection per contact), were estimated as global constants—that is, a single value for *k* and a single value for *q* were estimated over the entire study period (2011–2020), rather than being estimated month by month. Finally, the estimated optimal parameters *q* and *k* are substituted into the seasonally varying transmission rate matrix. Based on this, combined with the model-simulated monthly proportion of susceptible individuals S(t), the time-varying basic reproduction number $R_{0}\left(t\right)$ is calculated month by month by determining the spectral radius of the next-generation matrix (Equations (4)–(6)). This yields a monthly series of $R_{0}\left(t\right)$ reflecting the seasonal transmission potential.

## 3. Results

### 3.1. Age-Specific Force of Infection for Scarlet Fever in Shanghai

**Figure 4:**
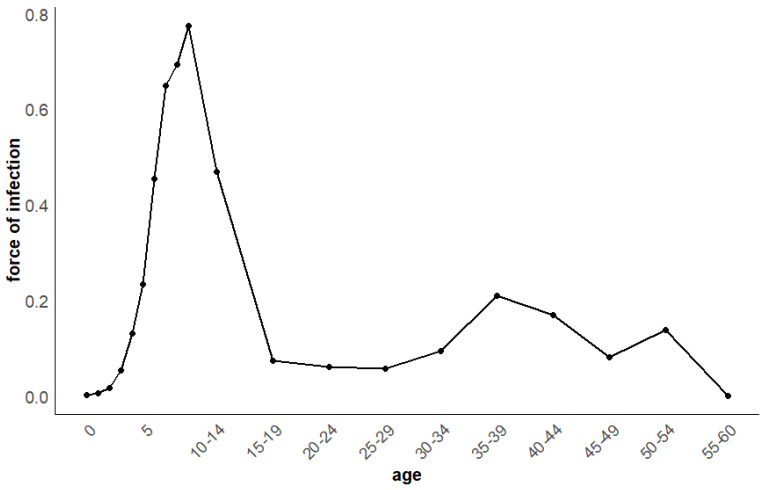
Age-specific force of infection (FOI) for scarlet fever in Shanghai, estimated using a catalytic model based on reported case data from 2011 to 2020. Estimates are shown for the first 20 age groups (0–59 years), with ages 0–9 years presented as single–year groups and ages 10–59 years as 5–year intervals. The FOI peaks sharply in children aged 7–9 years (64%–77% per year), followed by a secondary peak in adults aged 35–39 years (approximately 20% per year).

The FOI of scarlet fever across different age groups in Shanghai was estimated using catalytic modeling. The results indicate substantial variation in FOI among age groups. The highest FOI was observed in the 7–9 years age group, ranging approximately from 64% to 77%. The force of infection analysis (stratified into 26 age groups) revealed that adults aged 35–39 years had a relatively higher infection risk (approximately 20%) than other adult age groups. The lowest FOI was found in infants under 2 years old, at less than 1%. These findings highlight the distinct age-dependent risk pattern for scarlet fever transmission (Figure 4).

### 3.2. Seasonal Patterns and Drivers of Scarlet Fever Transmission in Shanghai

**Figure 5:**
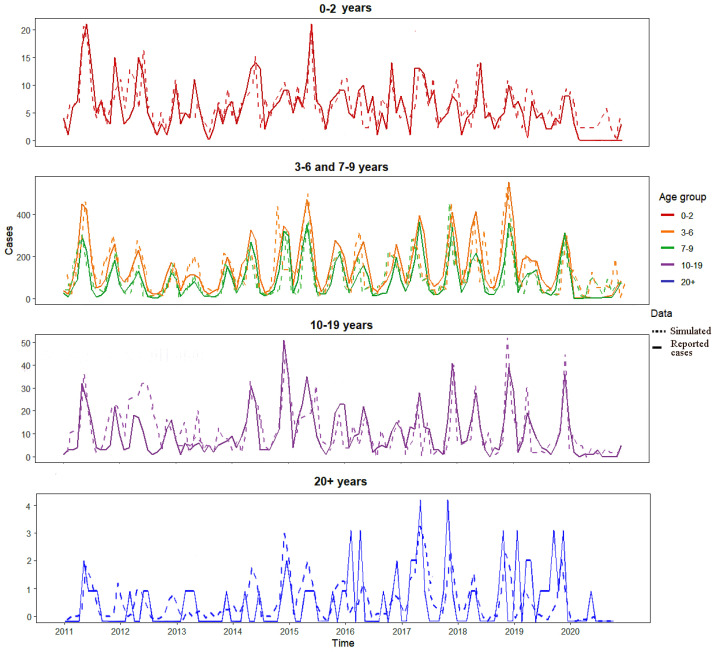
Comparison between observed and model-simulated monthly scarlet fever cases in Shanghai, 2011–2020, stratified by age group. For visual clarity, the original five age groups are combined into four panels: toddlers (0–2 years); adults (≥20 years); high-incidence childhood groups (3–6 years and 7–9 years, combined for visual clarity with separate lines for each group); adolescents (10–19 years). Simulated cases are generated from the age-structured SIR model with seasonal forcing (Equation (2)), using the estimated parameters *q* = 0.123 and *k* = 0.39. Solid lines represent observed cases; dashed lines represent simulated cases. The model successfully captures the characteristic bimodal seasonal pattern of scarlet fever, with peaks in May–June and November–December.

Using the contact rate matrix for Shanghai, based on the age-structured SIR model and the POMP inference framework described in Section 2.5, we estimated the following key parameters. The probability of infection per contact (*q*) for scarlet fever was estimated as 0.123 (95% CI: 0.12–0.13). The amplitude of seasonal forcing (*k*) affecting the transmission rate among school-aged groups was estimated at 0.39 (95% CI: 0.37–0.41), indicating that the increase in contact rates due to school openings raises the transmission rate by 39% above its mean level. The reported incidence of scarlet fever exhibits pronounced seasonal variation. These estimated parameters, *k* and *q*, were incorporated into an age-structured SIR model for simulation (Figure 5). The model achieved good agreement with the reported data across all age groups (Figure 5).

To evaluate the model fit, we calculated the Pearson correlation coefficient and the root mean square error (RMSE) between the simulated and observed monthly case counts for each age group. The RMSE is defined as $RMSE=\sqrt{\frac{1}{T}\sum_{t=1}^{T}{\left(S_{t}-R_{t}\right)}^{2}}$, where $S_{t}$ and $R_{t}$ are the simulated and reported cases at month *t*, respectively. The correlation coefficients between simulated and reported monthly cases ranged from 0.71 (20+ years) to 0.84 (3–6 years), with an overall correlation of 0.88 for all ages combined. The root mean square errors (RMSE) were 1.2, 5.7, 4.3, 4.1, and 1.1 cases per month for the 0–2, 3–6, 7–9, 10–19, and 20+ years age groups, respectively, reflecting the varying magnitude of incidence across groups (Table 2). These metrics indicate that the age-structured model with seasonal forcing adequately captures both the temporal dynamics and age-specific patterns of scarlet fever transmission.

### 3.3. Basic Reproduction Number

**Figure 6:**
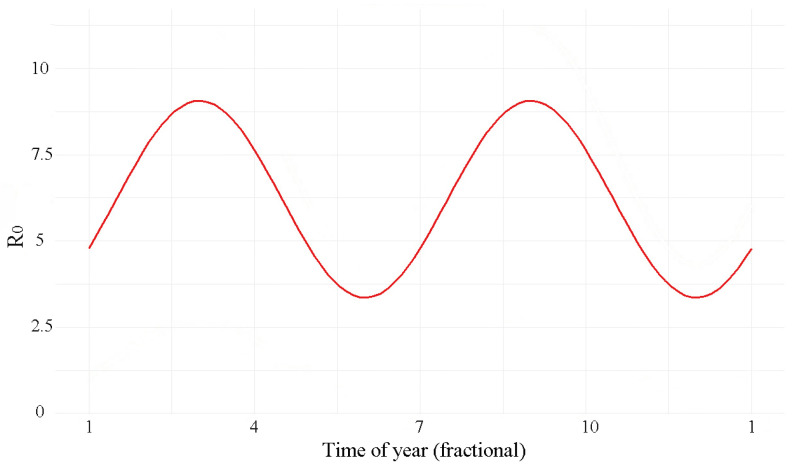
Time-dependent basic reproduction number. Monthly estimates are derived from the next-generation matrix (Equations (4)–(6)) using the seasonally varying transmission rate matrix β(t), (Equation (3)) and the estimated parameters *q* = 0.123 and *k* = 0.39. $R_{0}\left(t\right)$, exhibiting a pronounced biannual pattern, ranging from 3.02 to 8.83 (mean: 6.3), with peaks in March and September coinciding with school openings.

The classical next-generation matrix method typically employs a constant, time-invariant transmission rate matrix to describe the generation of new infections. However, in this study, the transmission rate β is a matrix characterized by seasonal variation (Equation (3)). To capture the dynamic changes in transmission, we calculated the time-varying basic reproduction number $R_{0}\left(t\right)$ by substituting the seasonally adjusted β matrix month by month into the core formula of the next-generation matrix (Equation (6)) and computing its spectral radius. As a result, the estimated basic reproduction number $R_{0}$ also exhibits corresponding seasonal fluctuations. Since the transmission rate peaks in March and September, applying the next-generation matrix method yields an estimated average time-varying basic reproduction number for the entire population in Shanghai from 2011 to 2020 that also shows two corresponding peaks. The estimated $R_{0}\left(t\right)$ varies between 3.02 and 8.83, with an average value of 6.3 (Figure 6).

## 4. Conclusions

Analysis of the age-specific force of infection (FOI) for scarlet fever in Shanghai from 2011 to 2020 indicated that the highest FOI occurred among 9-year-old children, within the broader 7–9-year age group. This pattern differs from measles, in which the FOI peaks in infants under 1 year of age [13], and from pertussis, where the highest FOI is observed around 6 years of age [16]. The average FOI across the 7–9-year age group ranged from 65% to 77%. However, due to the smaller susceptible population in this age range, reported case counts were lower than those in the 3–6-year age group. These findings suggest that the highest risk of scarlet fever infection in China is among younger primary school children. Therefore, prevention and control measures should prioritize this population. Among adults, individuals aged 35–39 years showed a relatively higher FOI, a pattern similar to that reported for pertussis [16]. This age group is likely to have more frequent contact with children—who themselves exhibit high FOI—than other adult age groups, which may explain their elevated infection risk. These FOI patterns motivate an age-structured modeling framework with seasonal forcing to quantify age-specific risks under varying contact patterns. Therefore, in the prevention and control of scarlet fever, greater attention should be focused on primary school children, especially those aged 7–9 during the back-to-school season. Parents should encourage children to maintain good hygiene practices, avoid contact with scarlet fever patients, and wear masks when visiting public places during peak seasons.

The age heterogeneity and seasonality of scarlet fever transmission in Shanghai are consistent with an underlying mechanism driven by age-specific contact patterns and their school-term variation, as encoded in our model assumptions. Among school-aged children and adolescents, contact rates vary substantially across the year (amplitude: 39%). This 39% amplitude implies that the transmission risk among school-aged children during term time is nearly 40% higher than during holiday periods. For a 7–9-year-old child in Shanghai, whose baseline FOI is approximately 70%, the instantaneous infection risk can approach near-complete levels at the peak of the school term. These findings provide a quantitative basis for timing non-pharmaceutical interventions: the greatest marginal benefit of measures such as hand hygiene campaigns, mask-wearing recommendations, and school absenteeism surveillance is expected in the early weeks of the spring (February–March) and autumn (September–October) semesters. Studies of varicella in England and Wales showed that holiday-related reductions in contact rates lowered transmission by 22–30% compared to term time [19], while research on measles in China indicated that school-term increases raised transmission by 31% [13]. Seasonal effects appear stronger in Chinese studies, with scarlet fever showing greater seasonal influence than other common childhood diseases. These results imply that school-driven contact patterns can induce season-wide variation in population transmission, reducing infection risk during holidays. Based on Shanghai’s transmission dynamics, the estimated basic reproduction number ($R_{0}\left(t\right)$) for scarlet fever ranges from 3.02 to 8.83. Although our estimate differs from some domestic studies that reported lower values [17,18,26], it aligns closely with historical estimates from pre-vaccination era Europe: 5.37–10.2 in Copenhagen and 6.4–7.7 in England and Wales [27,28]. The observed $R_{0}\left(t\right)$ range of 3.02–8.83 in Shanghai is therefore consistent with the intrinsic transmissibility of scarlet fever in densely populated urban settings with strong school-driven contact seasonality, rather than an artifact of our seasonal forcing specification.

This study employed an age-structured model to analyze the epidemic characteristics of scarlet fever in Shanghai. While the model accounts for heterogeneity in contact rates across different age groups, the seasonality was assumed to be identical for the three school-age groups, which contributed to some discrepancy between simulated and observed incidence. It should be noted, however, that the compartmental model adopted here is primarily intended to capture the dynamic features of disease transmission rather than to achieve precise prediction, as often emphasized in statistical models. The focus of an age-structured compartmental model lies in understanding how contact patterns across age groups shape epidemic behavior, making it particularly suitable for investigating the driving factors of transmission patterns in childhood infectious diseases. One potential limitation of our modeling framework is the assumption that seasonal forcing predominantly affects within-school-age-group transmission (i.e., $\beta_{22}$, $\beta_{33}$, $\beta_{44}$), while between-group contacts (e.g., $\beta_{23}$, etc.) are assumed to be non-seasonal. Although this simplification is supported by the strong assortative mixing patterns observed in the contact matrix and the high FOI in these groups, we acknowledge that it may not fully capture the complexity of school environments where cross-age interactions occur. However, given the monthly resolution of our data, this parsimonious specification was necessary to maintain the identifiability of the parameters. A limitation of this study is that we did not test alternative drivers of seasonality, such as temperature or humidity, which may affect respiratory infections. While our contact-driven model reproduces the observed age patterns and bimodal seasonality, other factors could also contribute. Future studies integrating contact data with environmental covariates are needed. Additionally, the contact matrix used in the secondary analysis assumed a uniformly distributed population, which also partly explains the deviation between simulated outputs and real-world data. Therefore, building on the current understanding of the seasonal pattern of scarlet fever, further refinement of age stratification and estimation of age-specific parameters constitute our next research objective.

## Figures and Tables

**Table 1:** Parameter values and estimated model parameters. Birth rate (ν) and mortality rate (μ) are from Shanghai statistical yearbooks [21], converted to monthly rates. Recovery rate (γ) corresponds to a mean infectious period of 6.25 days [17]. Aging rates ($\alpha_{i}$) are applied as discrete events at the start of each year. Per-contact infection probability (*q*) and seasonal amplitude (*k*) are estimated via particle filtering (see Section 2.5).

Parameter	Definitions	Units	Values	Sources
ν	Birth Rate	Month	0.00062	[21]
μ	Death Rate	Month	0.00071	[21]
γ	Recovery Rate	Month	4.8	[17]
$\alpha_{1}$	Aging Rate	Year	1/3	Assumed
$\alpha_{2}$	Aging Rate	Year	1/4	Assumed
$\alpha_{3}$	Aging Rate	Year	1/3	Assumed
$\alpha_{4}$	Aging Rate	Year	1/10	Assumed
$\alpha_{5}$	Aging Rate	Year	0	Assumed
*q*	Per-Contact Infection Probability	Dimensionless	Estimate	Estimate
*k*	Seasonality	Dimensionless	Estimate	Estimate

**Table 2:** Goodness-of-fit metrics for the age-structured model by age group, Shanghai. Pearson correlation coefficients and root mean square errors (RMSE, in cases per month) between model-simulated and observed monthly scarlet fever cases are shown for each age group. All correlation coefficients are statistically significant (*p* < 0.001).

Age Group	Correlation	RMSE (Cases/Month)
0–2 years	0.76	1.2
3–6 years	0.84	5.7
7–9 years	0.82	4.3
10–19 years	0.79	4.1
20+ years	0.71	1.1

## Data Availability

The data presented in this study are openly available in [CDC] at [https://www.chinacdc.cn/], reference number [20].
